# Sanhua decoction: Current understanding of a traditional herbal recipe for stroke

**DOI:** 10.3389/fnins.2023.1149833

**Published:** 2023-04-13

**Authors:** Lanlan Zheng, Linglei Meng, Huazheng Liang, Jiandao Yang

**Affiliations:** ^1^Department of Neurology, Shanghai Jiangong Hospital, Shanghai, China; ^2^Clinical Research Center for Anesthesiology and Perioperative Medicine, Shanghai Fourth People’s Hospital, School of Medicine, Tongji University, Shanghai, China; ^3^Translational Research Institute of Brain and Brain-Like Intelligence, Shanghai Fourth People’s Hospital, School of Medicine, Tongji University, Shanghai, China; ^4^Department of Anesthesiology and Perioperative Medicine, Shanghai Fourth People’s Hospital, School of Medicine, Tongji University, Shanghai, China; ^5^Monash Suzhou Research Institute, Suzhou Industrial Park, Suzhou, Jiangsu, China

**Keywords:** Sanhua decoction, ischemic stroke, composition analysis, pharmacological analysis, clinical efficacy

## Abstract

Both thrombolytic and endovascular therapies are optimal treatment options for patients with acute ischemic stroke, but only less than half of these patients can benefit from these treatments. Traditional Chinese medicine has a long history of successfully managing ischemic stroke using both herbal and physical therapeutics. Among herbal recipes, Sanhua decoction (SHD) is one of the classical prescriptions for ischemic stroke. The present review aimed to summarize evidence from both clinical and basic research to demonstrate its efficacy in managing ischemic stroke and the potential mechanisms underlying its therapeutic effects, which will provide evidence on the therapeutic effect of this herbal recipe and guide future studies on this recipe. SHD is composed of four herbs, *Rheum palmatum* L. [Polygonaceae], *Magnolia officinalis* Rehder & E.H.Wilson [Magnoliaceae], Citrus × aurantium L. [Rutaceae], Hansenia weberbaueriana (Fedde ex H.Wolff) Pimenov & Kljuykov [Apiaceae]. We found that the majority of clinical studies on SHD are case reports and they showed positive therapeutic effect of SHD on both acute and chronic ischemic stroke. There are over 40 bioactive compounds identified in SHD, but few experimental studies have examined their individual molecular mechanisms. As an extract of SHD, it improves neurological functions through suppressing inflammation, protecting the blood brain barrier from degradation, restoring the number of neural stem cells, inhibiting apoptosis and brain edema, scavenging oxygen free radicals, and regulating the brain-gut axis. These will lay the theoretical foundation for future studies on this prescription and its clinical application. Future research may need to confirm its clinical efficacy in large-scale clinical trials and to disentangle its bioactive compounds and their potential mechanisms.

## Introduction

Ischemic stroke is the most common cerebrovascular disease in clinical practice. Its incidence is steadily increasing with the improvement of modern living standards and the acceleration of the pace of life (Wang et al., 2019), and its onset of age is getting younger (Hankey, 2017). Hemiplegia, slurred speech, loss of consciousness, are the major manifestations (Zhang and Wu, 2017). Stroke remains one of the leading causes of death worldwide, with a high incidence, a high morbidity rate, and a high recurrence rate (Wang et al., 2019), especially in China where stroke is the leading cause of adult disability and death (Gbd 2016 Headache Collaborators, 2018). It poses heavy medical and financial burden to patients and their families, as well as the society (Wang W. et al., 2017; Pandian et al., 2018).

Studies have shown that stroke has complex etiologies and pathogenic mechanisms. Western medicine remains the first choice of treatments. Tissue derived plasminogen activator (tPA) is the only drug approved by FDA. It has an ideal time window of 4.5 h after symptom onset before starting intravenous infusion (Rangaraju et al., 2015). Emerging studies also support the use of endovascular thrombectomy. A few randomized clinical trials have extended the time window of endovascular thrombectomy from 6 to 24 h with the guidance of imaging results (Fransen et al., 2014; Pirson et al., 2021). However, the reality is that a decent proportion of ischemic stroke patients do not reach the hospital within the time window and others do not meet the criteria for thrombolysis or thrombectomy though they arrive at the hospital within the time window. These patients will be managed with secondary prevention measures leaving them with disabilities of various extents. Even in China where Traditional Chinese Medicine (TCM) was developed, few people received TCM therapies in the emergency room. One of the hurdles for the wide application of TCM therapies is that the therapeutic mechanisms of these herbal recipes or physical therapies are unclear though they have been empirically used for thousands of years. However, a large proportion of patients prefer to receive TCM therapies at the sequelae stage when western medicines have limited effects on their conditions.

Traditional Chinese Medicine has a long history of managing stroke with herbs or physical therapies, either to treat ischemic or hemorrhagic stroke (Li et al., 2016; Seto et al., 2016). Many literatures show that TCM treatment can prevent the exacerbation of this condition, significantly improve clinical outcomes of patients by promoting their functional recovery (Li et al., 2016; Seto et al., 2016; Bi, 2022). Recent studies have found that the addition of TCM recipes to conventional medicine at the acute stage of ischemic stroke displays superiority to pure conventional medicine, and the incidence of adverse events is low (Liu and Xiong, 2013). More importantly, many patients may have missed the time window for intravenous thrombolysis or endovascular thrombectomy, and they have no better options to choose but secondary prevention measures. In this particular respect, TCM treatments have displayed advantages over conventional medicines. Therefore, there is a surge of both clinical and basic research on the therapeutic effect and their underlying mechanisms of TCM recipes. Findings from these studies will demonstrate both clinical and mechanistic evidence of these recipes on ischemic stroke, and promote their use both in China and other countries (Zhang, 2019).

## TCM understanding of ischemic stroke

According to the TCM theory, ischemic stroke is closely related to accumulation of internal injury, deficiency of Qi (a form of energy which waxes and wanes in the body depending on health) and blood, excessive fatigue and lack of rest, emotional disturbance, unhealthy dieting habit, and obesity (Wang Y. et al., 2017). Pathogenic mechanisms mainly include external wind, extra heat, phlegm, blood stasis, and deficiency of Qi and blood (Si et al., 2019), which are closely related to the climate, emotional response, pressure, and other factors in daily life (Duan, 2017). These pathological factors contribute to the imbalance of Yin and Yang, dysfunction of Zang (solid organs) and Fu (hollow organs like guts, stomach), altered homeostasis of Qi and blood. As a result, ischemic stroke occurs (Li et al., 2019). Treatment of ischemic stroke should also be tailored based on the etiologies and pathogenic mechanisms of patients. Commonly used treatments mainly include nourishing Yin to expel the wind, clearing extra heat to protect the liver, invigorating Qi, improving blood circulation, cleansing blood stasis, dissipating extra heat and phlegm in internal organs, and restoring the consciousness (Li, 2018; Zhao and Zhou, 2021).

## Composition and application of Sanhua decoction

Sanhua decoction (SHD) first appeared in the book named “Su Wen Bing Ji Qi Yi Bao Ming Ji” (literally translated into Su Wen—collection of experience on how to live longer by understanding the pathogenic mechanisms and Qi) written by Liu (2007). He pointed out that ischemic stroke can be the result of dysfunction of a variety of organs. If it is due to the dysfunction of Fu (hollow organs), it is very likely to manifest with symptoms of both Fu and Zang (solid organs), like stasis of Qi and loss of consciousness. Treatments should aim to recanalize Fu (hollow organs) or to restore the consciousness (Chen et al., 2021). SHD is a representative prescription for stroke that recanalizes Fu. It has the effect of harmonizing Qi, blood, and body fluid, cleansing stasis in Fu through which the environment and the internal organs are connected (Zhao and Jie, 2016; Wang Y. et al., 2020).

Sanhua decoction is composed of four herbs, *Rheum palmatum* L. [Polygonaceae], *Citrus* × *aurantium* L. [Rutaceae], *Magnolia officinalis* Rehder & E.H.Wilson [Magnoliaceae], and *Hansenia weberbaueriana* (Fedde ex H.Wolff) Pimenov & Kljuykov [Apiaceae]. It is used for treating apoplexy of six meridians (Dong and Fang, 2005; Gong, 2007). These four herbs have been recorded in ancient books to effectively treat stroke (Song et al., 2016). “Yi Xue Wen Dui” (literally translated to Questions and Answers in Medicine) recorded that SHD is composed of Xiao Chengqi decoction and Qiang Huo [*Hansenia weberbaueriana* (Fedde ex H.Wolff) Pimenov & Kljuykov [Apiaceae]] (Gao, 1959). It is interpreted that *Rheum palmatum* L. [Polygonaceae] is the king herb, responsible for purging heat and the bowel; *Citrus* × *aurantium* L. [Rutaceae] is the minister herb, removing Qi stasis and retention of food in the bowel in addition to dissolving phlegm. These two herbs, when used together, can effectively eliminate extra heat, remove Qi stasis, and expel the retained food in the bowel. As a result, the gastrointestinal system is well-restored. *Magnolia officinalis* Rehder & E.H.Wilson [Magnoliaceae] is the assistant herb, specialized in removing Qi stasis and distention, and assists the other two herbs to recanalize the gastrointestinal system. These three herbs from Xiao Chengqi decoction are used for conditions manifesting with regurgitation of the gastrointestinal system. *Hansenia weberbaueriana* (Fedde ex H.Wolff) Pimenov & Kljuykov [Apiaceae] is the envoy herb, responsible for dispelling cold and wind, eliminating extra water, and ameliorating pain (Song et al., 2016). The combined use of these four herbs recanalizes meridians, purges Fu (hollow organs), tonifies Qi and blood, removes stasis, and restores the function of the brain and other organs (Fan et al., 2012b; Wang and Xie, 2013).

To search for studies on SHD, 4 English databases including PubMed, Web of Science, EMBASE, Cochrane Central Register of Controlled Trials, four Chinese databases including the Chinese National Knowledge Infrastructure, Wanfang Database, Chongqing VIP Database, and the Chinese Biomedical Database, and two clinical trial registration websites-the International Clinical Trials Registry Platform and the Chinese Clinical Trial Registry were searched up to March 30, 2022. Studies including randomized controlled trials, case control studies, reviews, or systematic reviews were included for analysis. Discrete searching strategies were used, including: “Sanhua decoction” or “Sanhua Tang” and “acute ischemic stroke” or “acute cerebral infarction” or “apoplexy” or “stroke” or “ischemic attack.” No language restriction was used. We found 5 articles from Pubmed, 7 from Web of Science, 0 from Cochrane database, 7 from EMBASE, 29 from CNKI, 34 from Wanfang Database, 10 from VIP Database, and 25 from Chinese Biomedical Database. Two out of seven articles written in English are published in Chinese but their titles are translated into English. Articles from four Chinese databases are overlapping with each other. In total, the number of articles directly related to SHD is 34, and partially related to SHD is 37. The latter includes studies on one herb or single or multiple bioactive compounds. Among these 34 articles, 12 are clinical studies.

## Clinical studies on Sanhua decoction

Sanhua decoction has a wide range of clinical applications. Many clinical studies have found that it has significant therapeutic effects on stroke without apparent adverse reactions.

Though there is a lack of randomized controlled trials on this herbal recipe, a number of Chinese studies did report its efficacy in managing ischemic stroke. It was reported that the efficacy of this recipe alone ranged from 83 to 95% for acute stroke patients compared with conventional western medicines excluding tPA and endovascular thrombectomy (Li and Dou, 2008; Yang et al., 2009; Liu, 2011; Wang C. et al., 2017). When SHD was used along with conventional western medicines, the therapeutic effect was even more significant (88.46 vs. 61.54%) (Wang, 2015). Among the patients, not only their NIHSS was improved, but also their Barthel indices (Yang et al., 2009; Liu, 2011). Apart from this, SHD significantly improved rheological parameters, including the decreased viscosity of the blood, decreased levels of fibrinogen, hematocrit (Yang et al., 2009), and TXB2 (Wang C. et al., 2017) when used in combination with conventional western medicines. In the meantime, plasma AT-III and 6-keto-PGF1α were increased after SHD treatment (Wang C. et al., 2017). In the largest cohort of 120 stroke patients, 60 received modified SHD. It was shown that neurological functions of 28 patients were completely restored, 20 significantly improved, 9 improved, and 3 unchanged. In comparison, the conventional western medicine group had 23 completely restored, 19 significantly improved, 8 improved, 10 unchanged. The difference between these two groups was statistically significant (Li and Dou, 2008), demonstrating the effectiveness of SHD in managing ischemic stroke.

In addition, SHD was found to be effective for chronic ischemic stroke patients with sequelae. Zhu and Guo (2000) reported that it improved neurological functions of ischemic stroke patients and prevented the recurrence of stroke after optimizing the dosage of the four herbs of SHD.

Apart from clinical studies using the standard SHD, a couple of studies modified this recipe based on patients’ conditions and also found that the modified recipes were also effective for ischemic stroke patients (Duan, 2005; Meng and Liu, 2007). For example, when *Conioselinum anthriscoides* ‘Chuanxiong’, *Angelica sinensis* (Oliv.) Diels [Apiaceae], *Acorus calamus* var. angustatus Besser [Acoraceae], and *Asarum sieboldii* Miq. [Aristolochiaceae] were added to the recipe, over 90% of patients with ischemic stroke showed improvement in their neurological functions (Meng and Liu, 2007). Similarly, another study added *Neolitsea cassia* (L.) Kosterm, *Pueraria montana* var. lobata (Willd.) Maesen & S.M.Almeida ex Sanjappa & Predeep [Fabaceae], *Mitragyna inermis* (Willd.) Kuntze [Rubiaceae], *Lycopus virginicus* L. [Lamiaceae], and *Tragia involucrata* L. [Euphorbiaceae] to the recipe, and 84.7% of the patients showed improvement in their neurological functions (Duan, 2005). There are other reports on the clinical use of SHD, but the number of patients was so small that they were not stated in this review. One example is the case report by Zhang and Ren (2002), where intractable hiccup due to stroke was alleviated by SHD.

## Formula analysis of SHD

### *Rheum palmatum* L. [Polygonaceae]

This herb is a commonly used one in TCM. It tastes bitter and has a cooling effect, specifically entering the spleen, stomach, large intestine, liver, and the pericardium meridians (Chen et al., 2019). It is known to remove bowel stagnation, clear extra water in the body, purge extra heat, cool the blood, clear blood stasis, and to detoxify toxins (Chinese Pharmacopoeia Commission, 2015). To be specific, this herb has a variety of pharmacological effects. It can regulate the function of the gastrointestinal system through anti-inflammatory and cholagogic effects, and increasing pancreatic secretion. It can also protect the cardiovascular system through regulating the metabolism of blood lipids, inhibiting the pathogenesis of atherosclerosis (Chen et al., 2019; Liu et al., 2020), scavenging free radicals (Heo et al., 2010; Song, 2019), and regulating the hemopoietic system. It improves the renal function, presenting with anti-inflammatory, antibacterial, antiviral, and anti-tumor effects (Du et al., 2018; Jin et al., 2020). In the central nervous system, it inhibits the production of nitric oxide and nitrosation or oxidation of proteins (Zhao et al., 2018), as well as the aggregation of platelets (Nemmar et al., 2015). As a result, it is empirically used to treat ischemic stroke.

### *Magnolia officinalis* Rehder & E.H.Wilson [Magnoliaceae]

This herb has been used in China for over 2,000 years. It is warm and bitter in nature, spicy in taste, and enters the spleen, stomach, lung, and the intestine meridians (Chinese Pharmacopoeia Commission, 2015). It mainly removes Qi stasis and stagnation, clears extra water and dispels distention, suppresses regurgitation, and pacifies asthma (Wei et al., 2019).

It is known for a number of therapeutic effects on the digestive, nervous, cardiovascular, and respiratory systems. It inhibits leukocyte infiltration to the brain under ischemia, production of free radicals (Liou et al., 2003) and TXB2 (Yu et al., 2016b), suppresses brain edema (Nemmar et al., 2015), and increases the content of dopamine, serotonin, 5-HIAA in the brain (Yang et al., 2017). Consequently, the cerebral blood flow is increased (Yu et al., 2016a), brain edema attenuated, and oxidative stress relieved. In addition, it has anti-inflammatory, analgesic, anti-bacterial, anti-tumor effects (Tan et al., 2020).

### *Citrus* × *aurantium* L. [Rutaceae]

This herb has a long history of clinical use. It first appeared in “Shen Nong Ben Cao Jing.” It tastes bitter, spicy, sour, and is slightly cold in nature. It enters the spleen and the stomach meridians. It is known to remove Qi stasis and bowel stagnation, to dissolve phlegm, and to ameliorate paralysis of internal organs (Heo et al., 2010). It is mainly used to treat bowel stagnation, distention and pain, diarrhea with tenesmus, constipation, phlegm stagnation, Qi stasis, and other conditions (Zhang et al., 2015).

Pharmacologically, this herb improves the function of the gastrointestinal tract, and exerts anti-tumor, anti-oxidation, anti-bacterial, and anti-inflammatory effects (Qu et al., 2017). In the central nervous system, it can ameliorate neuronal apoptosis and oxidative stress induced by the ischemia and reperfusion injury (Wang et al., 2017a), attenuate mitochondrial dysfunction (Wang et al., 2017b). It also suppresses levels of nucleotide-binding oligomerization domain 2 (NOD2), receptor-interacting serine/threonine kinase (RIP2), nuclear transcription factor-kappa B (NF-kB), matrix metalloproteinase-9 (MMP-9) and up-regulates claudin-5, and minimizes the infarct volume and edema. Consequently, neurological functions are improved (Bai et al., 2014; Wang K. et al., 2020).

### *Hansenia weberbaueriana* (Fedde ex H.Wolff) Pimenov & Kljuykov [Apiaceae]

This herb was recorded in “Lei Gong Pao Zhi Yao Xing Jie” (literally translated to analysis of herb processing and pharmacological effects) (Li, 1998). It has a Yang nature with a light smell and spicy and bitter taste, and can tonify and dissipate Qi, expel the cold through the skin. It enters the bladder and the kidney meridians, dispelling the cold and the wind from the skin, removing extra water in the body, recanalizing the merians, and exerting an analgesic effect (Shi and Shi, 2017).

Pharmacological studies have shown that this herb possesses anti-inflammatory, antipyretic, antioxidant, antibacterial, analgesic (Guo et al., 2019), and anti-hypoxia effects (Li et al., 2015). In the central nervous system, it has been shown to reduce viscosity of the plasma, and to inhibit platelet aggregation and thrombosis (Lv et al., 1981; Zhang et al., 1996). Therefore, it is widely used to treat cardiovascular, cerebrovascular, gynecological, and gastrointestinal diseases (Yang et al., 2022b).

## Dosage of Sanhua decoction and possible bioactive compounds

The dose of each herb in SHD was clearly recorded in the book named “Su Wen Bing Ji Qi Yi Bao Ming Ji” by Liu (2007). Converted to current units, the dose of each herb equals to 30.5 g. They are boiled in 2,831.4 ml water and only half of the water is retained after being boiled twice. This water extract will be drunk within a day. Changes of the patients’ conditions will be closely monitored and drinking of SHD is ceased if diarrhea occurs (Zhang X. et al., 2022). Though this herbal recipe has been empirically used in clinical practice for centuries, no human pharmacokinetic research has been conducted. Little information is available to characterize this herbal recipe in this respect.

## Possible bioactive compounds

With the assistance of the rat middle cerebral artery occlusion (MCAO) model and the network pharmacology technique, correlations between active compounds, compound targets and signaling pathways were described. These targets or compounds were tested *in vivo* for verification, aiming to reveal the therapeutic mechanisms of SHD in managing ischemic stroke. Forty active compounds and 47 direct target genes were identified, indicating that this herbal recipe plays a pharmacological role in the treatment of ischemic stroke through multiple targets. Among the purified compounds, emodin anthrone, isopropamidine, and scopoletin were identified as key bioactive compounds. Numerous targets, including interleukin-6 (IL-6), amyloid precursor protein (APP), protein kinase B (AKT1), and vascular endothelial growth factor A (VEGFA) were considered to be major targets (Yang et al., 2022b). Multiple signaling pathways including endocrine resistance, estrogen, tumor necrosis factor (TNF), advanced glycation endproducts/receptor for advanced glycation endproducts (AGEs/RAGE), and microRNAs were regulated by SHD. As a result, ischemic injury and inflammatory reactions were attenuated (YingHuang et al., 2022).

In another study, 78 shared targets by ischemic stroke and SHD were identified through the network pharmacology technique. Based on these targets, 9 compounds targeting over 10 target genes were identified and they might be the key bioactive molecules of SHD. These included apigenin, luteolin, nobiletin, naringenin, β-sitosterol, emodin, tetra-methoxylluteolin, isosinensetin, and tangeretin (Zhang W. et al., 2022). In another study, phenethyl ferulic acid ester and (-)-bornyl ferulic acid ester were found to be the active compounds which can inhibit platelet aggregation (Zhang and Shen, 2008).

A similar study found that SHD had 24 key bioactive compounds for stroke and some of them showed potential to become medicines, including hesperidin, cedar acid, houpulin M, 6′-*O*-methylhonokiol, isomagnolol, syringaldehyde, and vanillic acid. These compounds mainly interact with 19 targets including APP, heat shock protein 90 alpha (HSP90AA1), recombinant mothers against decapentaplegic homolog 4 (SMAD4), argininosuccinate synthase 1 (ASS1), elastin (ELN), general transcription factor II-I (GTF2I), LIM domain kinase 1 (LIMK1), transducin (beta)-like 2 (TBL2), Von Hippel-Lindau (VHL), nuclear transcription factor-kappa B 2 (NFKB2), Jagged 1 (JAG1), Nuclear Receptor Subfamily 3 Group C Member 1 (NR3C1), tumor protein P53 (TP53), and MLX interacting protein-like (MLXIPL), etc. Seven core signaling pathways were identified and they were dominantly related to the anti-inflammatory effect of SHD, such as the interleukin-4 (IL-4) and interleukin-13 (IL-13) mediated signaling pathways (Yang et al., 2022a).

Aloe-emodin, rhein, emodin, chrysophanol, and physcion are in the same category of rhubarb aglycone. They were shown to improve neurological functions of MCAO rats through attenuating neuronal apoptosis, scavenging free radicals, suppressing nitric oxide mediated cytotoxicity and neuroinflammation, as well as preventing platelet aggregation (Li et al., 2003, 2005; Guan et al., 2014).

## Pharmacological mechanisms of SHD in treating ischemic stroke

Studies have found that SHD is an effective therapy for acute ischemic stroke without apparent adverse reactions (Yang et al., 2009; Liu, 2011). However, mechanisms underlying the therapeutic effect of SHD are still unclear. A summary of the known mechanisms was listed below.

## The anti-inflammatory effect of SHD

After cerebral infarction, a large amount of proinflammatory cytokines are released, among which IL-6 and TNF-α are the major players. They are used as biomarkers for the recurrence of vascular events (Boehme et al., 2016). IL-6 induces brain injury and hippocampal neuronal necrosis through activating the *N*-methyl-D-aspartate (NMDA) receptor and up-regulating the c-Jun N-terminal kinase (JNK) (Armstead et al., 2019). SHD can significantly attenuate swelling, degeneration, and necrosis of neural cell, as well as infiltration of inflammatory cells caused by cerebral ischemia, and can significantly reduce the expression of interleukin-1β (IL-1β) and intercellular adhesion molecule 1 (ICAM-1) (Wu et al., 2009; Fan, 2010; Fan et al., 2011). Emodin is likely to be the bioactive compound for this effect as it was shown to downregulate NF-κB and ICAM1 in MCAO rats (Wu et al., 2009). In network pharmacology studies stated above, IL-6, TNF-α, NF-κB, AGEs/RAGE, and other targets were identified, suggesting that SHD may target these genes or their proteins to take the therapeutic effect (Yang et al., 2022b; Zhang W. et al., 2022; Zhang X. et al., 2022). In addition, another study reported seven major signaling pathways targeted by SHD, including the NF-κB mediated signaling pathway where TNF receptor associated factor 6 (TRAF6) is involved, the NF-κB signaling pathway activated through TGF-beta activated kinase 1 (TAK1) phosphorylation and the Ikappa B kinases (IKKS), IL-4, and IL-13 mediated signaling pathways (Yang et al., 2022a). In another network pharmacology study, the targets of emodin were estimated, which included caspase 3, prostaglandin-endoperoxide synthase 1 (PTGS1), TNF, matrix metalloproteinase 9 (MMP9), protein kinase C epsilon (PRKCE), prostaglandin-endoperoxide synthase 2 (PTGS2), tumor protein 53 (TP53). Among them, caspase 3, PTGS1, TNF, and MMP9 are associated with inflammation (Jia et al., 2021). *In vivo* experiments also confirmed the decreased expression of IL-6 and TNF-α in one of these studies (Zhang W. et al., 2022). Fu et al. (2020) reported that SHD significantly improved neurological functions and reduced the expression of p-tau. The latter is a pathogenic protein involved in neurodegeneration, and triggers neuroinflammation in Alzheimer’s disease models. In another study on dogs with global ischemia-reperfusion injury, honokiol has been shown to attenuate the increased level of thromboxane B2 (TXB2), but it has no impact on the level of endothelin and NO. Surprisingly, whether neurological functions were improved by this compound was not reported in this study (Yu et al., 2016b). Another study used model rats of the ischemia-reperfusion injury and rats with spontaneous hypertension which are prone to develop stroke. They found that honokiol significantly increased the production and release of NO by endothelial cells, which mediates the dilation of blood vessels (Liu et al., 2016). Whether other bioactive compounds have the same effect is unknown.

## Inhibition of degradation of the blood brain barrier

Destruction of the blood-brain barrier (BBB) plays an important role in the occurrence and development of neurological dysfunction in ischemic stroke (Yang et al., 2019; Li et al., 2022). Fan (2010) showed that SHD could significantly reduce cerebral edema, increase the expression of zonula occludin-1 (ZO-1) in rats with the cerebral ischemia-reperfusion injury, and significantly reduce the content of S100β protein in the serum of these rats. When BBB is open or broken, S100β protein can reach the peripheral blood. This protein protects BBB in rats with the cerebral ischemia-reperfusion injury, which may be achieved by up-regulating the expression of ZO-1 protein, a linker protein between transmembrane proteins and cytoskeleton proteins (Li et al., 2022).

Matrix metalloproteinases (MMPs) are involved in neuronal injury after cerebral ischemia (Cuadrado et al., 2009; Fan et al., 2012a). Fan (2010) showed that SHD could significantly ameliorate neurological deficits of rats with the cerebral ischemia-reperfusion injury and attenuate the swelling, degeneration, and necrosis of neural cells. The level of MMP-9 mRNA and MMP-9 protein was also significantly reduced. Wang et al. not only found the decreased expression of MMP-9 in MCAO rats, but also decreased expression of nucleotide oligomerization domain-like receptors 2 (NOD2), receptor-interacting protein 2 (RIP2), and NF-κB, which are all regulators of the expression of proinflammatory genes, after pretreatment with naringenin. In addition, naringenin upregulated the expression of claudin-5 and consequently decreased the permeability of the BBB (Bai et al., 2014). Another study showed that SHD can upregulate the expression of krüppel-like factor 2 (KLF2), suppress the expression of thrombomodulin and eNOS, sustaining the integrity of the endothelial cells and maintaining their physiological functions (Wang et al., 2022).

Whether other compounds identified in SHD can protect the integrity of the BBB still needs investigation.

## Protection of neural stem cells and inhibition of apoptosis

Two studies reported the therapeutic effect of SHD in the rat model of the ischemia-reperfusion injury and found that this herbal recipe restored the number of endogenous neural stem cells. Not only BrdU positive and doublecortin positive cells, but also BrdU positive and GFAP positive cells were increased. Stem cell migration and differentiation was promoted by SHD, which was closely related to the improvement of neurological functions (Li, 2015; Fu et al., 2020). However, which bioactive compounds impart this effect is unknown. Phytochemicals are recognized by their multi-target characters (Khan et al., 2020). One herb contains thousands of chemicals and a number of them can protect or augment neurogenesis in the brain, like Egb761 (a chemical present in ginkgo) and resveratrol (present in grape products) (Jahan et al., 2018). Another study on bone marrow derived stem cells found that rhubarb aglycone, a bioactive compound from Rhubarb, increased the level of nerve growth factor (NGF) and glial derived neurotrophic factor (GDNF) after transplanting the stem cells to the brain of MCAO model rats (Li et al., 2008). This suggests that rhubarb aglycone might have positive effect on neural stem cells. The PI3K-Akt pathway is an important one in the development of ischemic brain injury (Samakova et al., 2019). It regulates many cellular functions, such as cell survival, autophagy, protein synthesis, and glycolysis (Xie et al., 2019). Phosphorylation of Akt increases the level of an anti-apoptotic protein B-cell lymphoma 2 (Bcl2). Recent studies reported that Akt phosphorylation enhanced nuclear translocation of the nuclear factor E2-related factor 2 (Nrf2), and phosphorylation of the cyclic adenosine monophosphate response element binding protein (CREB), a survival regulatory protein, and protected against cerebral ischemia (Zhang et al., 2018, Zhang X. et al., 2022). A study on the rhubarb extract showed that it significantly attenuated the increase in apoptosis, caspase-3, BCL2-associated X (Bax) in the MCAO model and increased the level of Bcl-2, suggesting that this extract might take effect through inhibiting the apoptosis pathway (Tang et al., 2020). In a study by Wang et al. (2017a), naringenin was found to prevent neuronal apoptosis and to suppress translocation of Nrf2 from the cytoplasm to the nucleus in MCAO model rats (Wang et al., 2017a). Whether other compounds are able to protect neurons from apoptosis still needs further investigation.

## Inhibition of brain edema

Aquaporin 4 (AQP4) is a functional regulator of astrocytes and a common water-conducting membrane integrin channel in the brain. Through regulating the inflow and clearance of cerebral water, AQP4 is involved in the development of cerebral edema and pathogenesis of various neurological conditions (Suzuki et al., 2020). In the study by Lu et al. (2015) SHD significantly alleviated neurological deficits after the cerebral ischemia/reperfusion injury and reduced the expression of AQP4. Through injecting lentivirus-mediated AQP4-siRNA into the ventricle of rats before inducing MCAO, decreased expression of AQP4 in the ipsilateral hippocampus and attenuated cerebral edema was found after modeling MCAO. Therefore, SHD can reduce the water content of the brain and effectively ameliorate the permeability of BBB.

Sodium channels are key to control the transmission of electrical signals in the nervous system, and its abnormality contributes to the development of cerebral infarction. In a study by Dai et al. (2011), they found that SHD significantly reduced the volume of cerebral infarction in rats and increased the expression of Nav1.1 mRNA. The latter resulted in reduce sodium influx and protection of neurons. Neuroinflammation is often accompanied by infiltration of inflammatory cells from the blood and increased levels of proinflammatory cytokines. The latter can injure the endothelial cells and activate microglia as well as astrocytes through multiple signaling pathways. One of the consequences is brain edema. Therefore, bioactive compounds present in SHD that attenuate inflammation may alleviate brain edema as well (Wu et al., 2009; Fan, 2010; Bai et al., 2014; Yu et al., 2016b).

## Scavenging oxygen free radicals

Reactive oxygen species (ROS) are important players in the development of ischemic stroke, especially during the ischemia-reperfusion injury. Therefore, scavenging or neutralizing ROS is one of the potential therapies for ischemic stroke. In the body, the superoxide dismuatase (SOD) is a cytoplasmic antioxidant enzyme that catalyzes the reaction with free radicals and scavenges them. SHD was reported to increase the content of SOD in rats with the cerebral ischemia-reperfusion injury when administered intragastrically. In the meantime, the content of malonaldehyde (MDA), a product of lipid peroxidation, was significantly decreased (Tang et al., 2008), indicating that SHD takes its effect partially through suppressing lipid peroxidation and accelerating the scavenging of ROS. In another study, honokiol, a compound present in Magnolia officinalis, was found to protect the brain tissue during the ischemia-perfusion injury through suppressing neutrophil infiltration and lipid peroxidation evidenced by the reduced level of MDA (Liou et al., 2003). Naringenin significantly decreased the production of ROS through activating the Nrf2/antioxidant response element signaling pathway (Wang et al., 2017b). Whether other compounds have this effect is still unknown.

## Potential involvement of the microbiota-brain-gut axis

The microbiota-brain-gut axis has been widely acknowledged to be involved in a large variety of disorders. Emerging studies have shown that the brain regulates the digestive system through not only the sympathetic, parasympathetic nervous system, but also through the hypothalamus-pituitary-adrenal gland axis as well as the endocrine system. Vice versa, the gut impacts the brain not only through neural transmission *via* the vagus nerve to upper brain regions, but also through diverse signals, including intestinal peptides like cholecystokinin and vasoactive intestinal peptide, small molecular weight compounds like dopamine, serotonin, and metabolites of bacteria in the gut. Both the nervous, endocrine, and immune systems are involved in this regulation. The detailed molecular mechanisms of these neural pathways, compounds, and immune cells as well as mediators in the pathogenesis of neurological conditions have been comprehensively reviewed by many researchers (Cryan et al., 2019; Agirman et al., 2021; Socała et al., 2021; Barrio et al., 2022).

In TCM, 6 Yang meridians are traveling through the brain, indicating their involvement in the pathogenesis of brain diseases. Among these six meridians, the colon meridian is responsible for the expelling of the remaining substance after absorption in the digestive system. The normal function of the colon meridian ensures that the brain receives supply of nutrients and essential molecules from the digestive system. When stroke occurs, the colon will be impacted more or less. One of the eight therapeutic strategies in TCM is catharsis. SHD, a recipe containing rheum palmatum, can recanalize the gut through purging the digestive tract. This not only removes the food residue retained in the gut, but also attenuates inflammation, and improves neural functions (Liu et al., 2005). It is reasonable to expect that the purge of the digestive tract will significantly alter the composition of the microbiota in it and subsequently the metabolites of bacteria, the interaction between bacteria and the gut epithelial cells. Eventually, neurological functions will be altered. However, few studies have examined this type of molecular mechanisms underlying the therapeutic effect of SHD. In the ischemia rat model, Fan et al. found that intragastric administration of SHD in aged rats increased the activities of Na^+^-K^+^- and Ca^2+^-ATPases (Fan et al., 2009), protecting the gastrointestinal tissue from injury caused by acute stroke. In another study, anthraquinone glycoside, a compound identified in rheum palmatum, was found to alleviate the ischemia-reperfusion injury and to increase the activity of SOD. In the meantime, Escherichia coli and enterococci were suppressed, lactobacillus and bifidobacterial were increased (Yu et al., 2019). In the thesis of Li (2012), it was found that citrus aurantium significantly attenuated inflammation in the mucosa of the stomach due to acute ischemic stroke, which might be related to reduced levels of gastrin, motilin, and vasoactive intestinal peptide. In the same model, suspension of citrus aurantium was shown to ameliorate inflammation in both the stomach and the small intestine, with even better results when nimodipine was used together. The expression of substance P (SP), connexin 43 (Cx43), C-kit in the stomach and the intestine was decreased along with a reduced number of interstitial Cajal cells, suspension of citrus aurantium plus nimodipine significantly reversed these changes, indicating that citrus aurantium is able to protect the mucosa of the digestive tract and restore its functions after ischemic stroke (Qiu, 2014). It is possible that SHD might take its therapeutic effect partially through regulating the brain-gut axis.

## Conclusion and outlook

**FIGURE 1:**
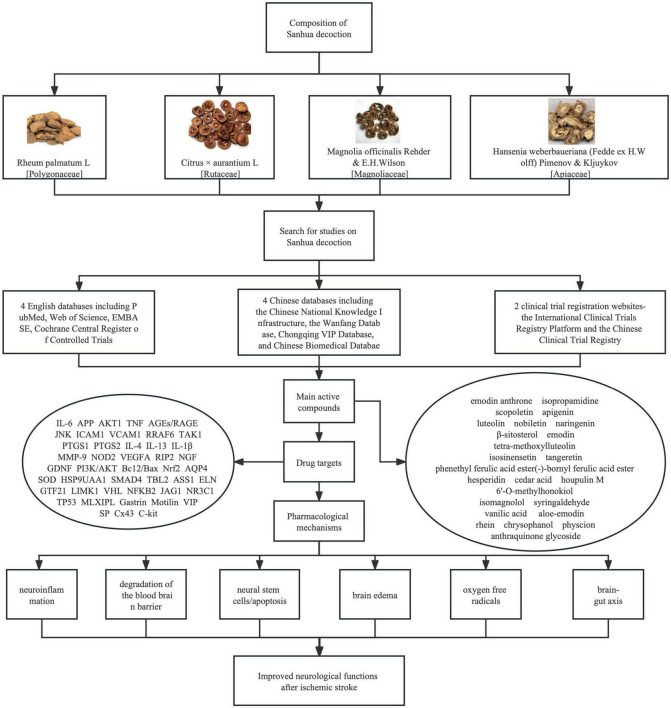
Pictures of four herbs constituting Sanhua decoction (SHD), searching strategies for articles on SHD and bioactive compounds identified from SHD, molecular mechanisms underlying their therapeutic effect on ischemic stroke. Both English and Chinese databases were searched for publications on SHD. Bioactive compounds were summarized from network pharmacology studies. These compounds can improve neurological functions of ischemic stroke patients through suppressing neuroinflammation, ameliorating, or preventing the degradation of the blood brain barrier, protecting neural stem cells and reducing apoptosis, preventing or mitigating brain edema, removing oxygen free radicals, and regulating brain-gut axis.

The present review summarized evidence supporting the therapeutic effect of SHD in managing ischemic stroke and its potential therapeutic mechanisms (Figure 1). There are a few possibilities to explain the potential advantages of SHD over western medicines in managing ischemic stroke patients. Firstly, SHD can be used beyond the time window for intravenous thrombolysis or endovascular thrombectomy, even at the chronic stage of ischemic stroke. This might be related to the anti-inflammatory, anti-apoptosis and other therapeutic mechanisms listed above. It is widely acknowledged that free radical scavengers like edaravone can effectively attenuate neurological deficits. The bioactive compounds isolated from SHD like naringenin and honokiol might have similar effects at the acute stage of ischemic stroke. Secondly, SHD contains multiple bioactive compounds which are targeting multiple pathways that are responsible for pathological changes after ischemic stroke. The synergistic effect of these compounds may augment their individual therapeutic effect. For example, the anti-inflammatory and free radical scavenging effects of SHD may more significantly alleviate brain edema than suppressing neuroinflammation alone or scavenging free radicals alone. This might be confirmed by applying different combinations of bioactive compounds to ischemic stroke models and even through randomized clinical trials. Thirdly, no specific medications have been developed so far to treat ischemic stroke and this is due to the lack of insight into the pathogenesis or mechanisms of ischemic stroke. There might be potential mechanisms that we have not discovered yet. SHD or other herbal recipes might have compounds that can target these mechanisms.

It is found from clinical practice that the therapeutic effect of TCM treatment based on syndrome differentiation is often better than that of Western medicine, and the incidence of adverse reactions is lower. The combination of SHD with conventional Western medicine in the treatment of stroke can effectively improve clinical efficacy, and is of great significance to ensure the long-term quality of life of patients. However, strict attention should be paid to drug interactions when Chinese and western drugs are used together. At present, large-scale, multi-center randomized, double-blind, controlled clinical trials on SHD are still lacking. In the future, it is necessary to reinforce the training of research design and clinical research capabilities of traditional Chinese medicine researchers, constantly explore opportunities to integrate Chinese and western medicine in managing a variety of medical conditions. The aim is to accelerate the recovery of these patients and to improve their quality of life.

## Author contributions

JY and HL conceived the study and revised the manuscript. LZ and LM searched the literatures and prepared the draft of this manuscript. All authors contributed to the article and approved the submitted version.
